# Donor-Acceptor Block Copolymers: Synthesis and Solar Cell Applications

**DOI:** 10.3390/ma7043274

**Published:** 2014-04-22

**Authors:** Kazuhiro Nakabayashi, Hideharu Mori

**Affiliations:** Department of Polymer Science and Engineering, Graduate School of Science and Engineering, Yamagata University, 4-3-16, Jonan, Yonezawa 992-8510, Japan; E-Mail: h.mori@yz.yamagata-u.ac.jp

**Keywords:** donor-acceptor block copolymer, conjugated polymer, cross-coupling reaction, electron-transporting material, nanomorphology, organic photovoltaic

## Abstract

Fullerene derivatives have been widely used for conventional acceptor materials in organic photovoltaics (OPVs) because of their high electron mobility. However, there are also considerable drawbacks for use in OPVs, such as negligible light absorption in the visible-near-IR regions, less compatibility with donor polymeric materials and high cost for synthesis and purification. Therefore, the investigation of non-fullerene acceptor materials that can potentially replace fullerene derivatives in OPVs is increasingly necessary, which gives rise to the possibility of fabricating all-polymer (polymer/polymer) solar cells that can deliver higher performance and that are potentially cheaper than fullerene-based OPVs. Recently, considerable attention has been paid to donor-acceptor (D-A) block copolymers, because of their promising applications as fullerene alternative materials in all-polymer solar cells. However, the synthesis of D-A block copolymers is still a challenge, and therefore, the establishment of an efficient synthetic method is now essential. This review highlights the recent advances in D-A block copolymers synthesis and their applications in all-polymer solar cells.

## 1. Introduction

**Figure 1.**
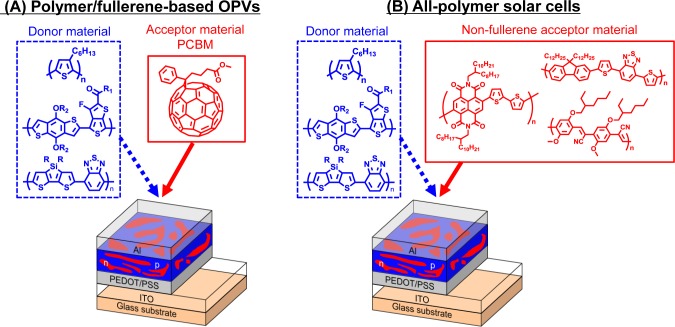
Device architecture of (**A**) polymer/fullerene organic photovoltaics (OPVs) and (**B**) all-polymer solar cells.

**Figure 2.**
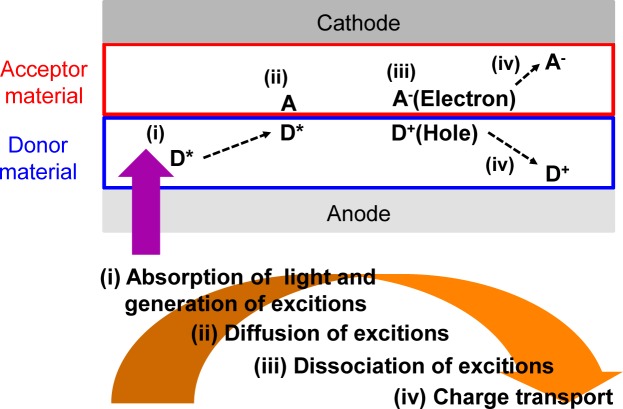
Four fundamental steps for the energy conversion process in solar cells; (i) absorption of light and generation of excitons; (ii) diffusion of the excitons; (iii) dissociation of the excitons; and (iv) charge transport and charge collection.

In recent years, organic photovoltaics (OPVs) based on conjugated polymeric materials have received considerable attention, because of their advantages, such as being low cost, light weight, flexible and having a facile large-scale fabrication, compared to silicon-based solar cells [1–6]. To date, polymer/fullerene (fullerene-based) OPVs, in which the active layers are composed of hole-transporting (*i.e*., donor (D)) polymeric materials and electron-transporting (*i.e.*, acceptor (A)) fullerene derivatives, have achieved power conversion efficiencies (PCEs) of over 10% (Figure 1A) [7]. Four fundamental steps of the energy conversion process are as follows: (i) absorption of light and generation of excitons in the donor domains; (ii) diffusion of the excitons to the donor-acceptor (D-A) interfaces (the exciton diffusion lengths before recombination are *ca*. 10–20 nm); (iii) dissociation of the excitons and generation of charge; and (iv) charge transport and charge collection (Figure 2).

For the realization of an efficient photoelectric conversion process, the formation of well-defined nanostructures in active layers is essential. Accordingly, block copolymers can be considered to be one of the promising candidates for OPV applications, because their nanostructures (e.g., lamellar, cylindrical, gyroid) can be controlled by adjusting the polymer structure [8–11]. The research groups of Yokozawa *et al.* [12] and McCullough *et al.* [13–15] developed the controlled polymerization of regioregular poly(3-hexylthiophene) by Grignard metathesis (GRIM) polymerization separately; since then, a number of regioregular polythiophene-based donor-type block copolymers have been developed [16–21]. Furthermore, detailed investigations have been carried out on the nanomorphology derived from their well-defined polymer structures (*M*_w_/*M*_n_ ~ 1.1–1.3) and their applications as donor materials in OPVs. For example, poly(3-hexylthiophene)-*block*-poly(3-(2-ethylhexyl)thiophene) [22], poly(butylthiophene)-*block*-poly(octylthiophene) [23,24] and poly(4-vinyltriphenylamine)-*block*-poly(3-hexylthiophene)-*block*-poly(4-vinyltriphenylamine) [25] were found to exhibit clear lamellar phase separation by atomic force microscopy (AFM) and transmission electron microscopy (TEM) observations. Furthermore, the use of poly(4-vinyltriphenylamine)-*block*-poly(3-hexylthiophene)-*block*-poly(4-vinyltriphenylamine) as a surfactant in the poly(3-hexylthiophene) (P3HT)/[6,6]-phenyl C_61_ butyric acid methyl ester (PCBM) OPV system enhanced PCE and long-term performance [26]. These results demonstrate that block copolymers can successfully function as OPV materials.

Quite recently, block copolymers composed of both donor and acceptor segments (D-A block copolymers) have been of interest, because of their potential applications as acceptor materials instead of fullerene derivatives in OPVs [27–31]. In contrast to donor materials, the fullerene derivative, PCBM, is the only conventional acceptor material for OPVs. One of the major reasons for using PCBM is its high electron mobility (*ca.* 0.002 cm^2^/V s). However, there are also considerable drawbacks for use in OPVs: (i) negligible light absorption in the visible-near-IR regions; (ii) less compatibility with donor polymeric materials; and (iii) high cost for synthesis and purification. The investigation of non-fullerene acceptor materials that can potentially replace PCBM in OPVs is, thus, increasingly necessary, which gives rise to the possibility of fabricating all-polymer (polymer/polymer) solar cells (Figure 1B). All-polymer solar cells offer potential advantages over conventional fullerene-based OPVs, such as more efficient light absorption due to the acceptor polymer and relatively high open-circuit voltages [32]. Although D-A block copolymer characteristics, like electron-transporting properties derived from acceptor blocks, broad light absorption and nanomorphology derived from the D-A block structure, can be considered to be ideal for use as OPV acceptor materials, the number of D-A block copolymers is still limited, because of their synthetic difficulties. However, academic interest in the synthetic methodology of D-A block copolymers and the potential application of these polymers in all-polymer solar cells motivated us to review the work. In this review, the recent advancements in D-A block copolymer synthesis and their applications for all-polymer solar cells are highlighted.

## 2. Block Copolymers with Acceptor Pendant Units

Bock copolymers with acceptor pendant units were synthesized using one of the following two techniques: (i) synthesis of block copolymers using monomers with acceptor pendant units; and (ii) synthesis of block copolymer precursors, followed by the post-modification to incorporate acceptor pendant units.

**Figure 3.**
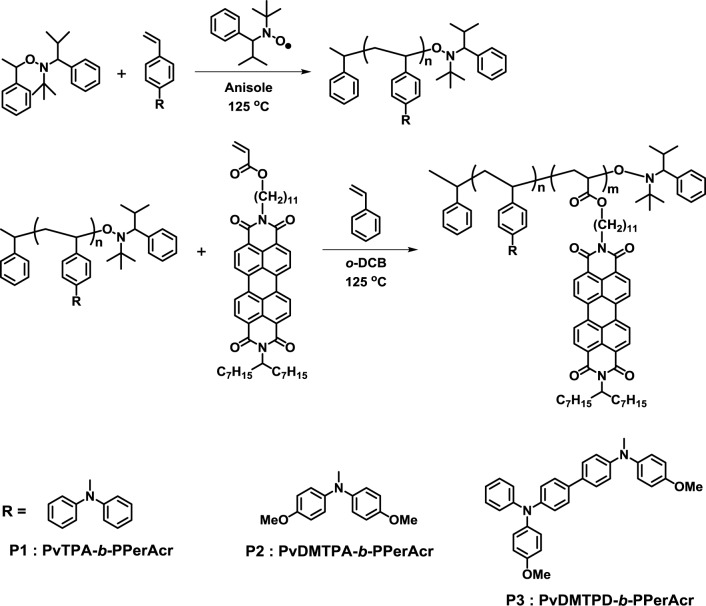
Synthesis of **P1**–**P3**.

In 2006, Thelakkat *et al.* reported the synthesis of poly(bisphenyl-4-vinylphenylamine)-*block*-poly(perylene diimide acrylate) (PvTPA-*b*-PPerAcr, **P1**), poly(bis(4-methoxyphenyl)-4’-vinylphenylamine)-*block*-poly(perylene diimide acrylate) (PvDMTPA-*b*-PPerAcr, **P2**) and poly(*N,N’*-bis(4-methoxyphenyl)-*N*-phenyl-*N’*-4-vinylphenyl-(1,1’-biphenyl)-4,4’-diamine)-*block*-poly(perylene diimide acrylate) (PvDMTPD-*b*-PPerAcr, **P3**) using the monomer with acceptor pendant units through nitroxide-mediated polymerization (NMP) (Figure 3) [33,34]. The molecular weight of polymers and PPerAcr acceptor block contents were controlled using macroinitiators with various molecular weights. Depending on the molecular weight of macroinitiator and PPerAcr block content, wire-like or worm-like nanostructures with nano-size domains were observed in TEM measurements. They also demonstrated that **P1** could form lamellar and vesicle-like nanostructures by solvent vapor annealing at low temperatures [35].

**Figure 4.**
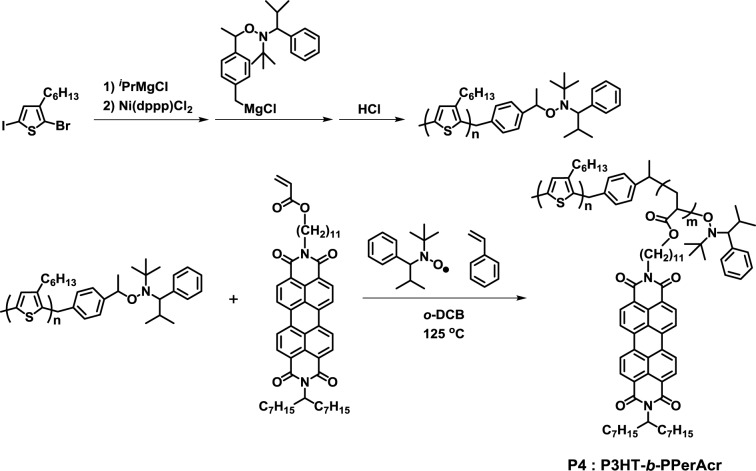
Structure of **P4**.

In 2008, the synthesis of crystalline-crystalline poly(3-hexylthiophene)-*block*-poly(perylene bisimide acrylate) (P3HT-*b*-PPerAcr, **P4**) by NMP using the PPerAcr monomer was reported (Figure 4) [36]. End-functionalized P3HT was successfully prepared by GRIM polymerization. **P4** with molecular weight (PPerAcr acceptor block contents) in the range of 16,100 (53.2%)–24,800 (81.4%) was successfully synthesized by varying the reaction time and the ratio of the P3HT-based macroinitiator and PPerAcr monomer. Among them, **P4** with *M*_n_ = 16,100 (53.2%) exhibited the cylindrical nanostructure of PPerAcr in P3HT matrix by scanning electron microscopy (SEM) observation. This was the first report on clear microphase separation in a fully functionalized block copolymer consisting of two crystalline blocks. Furthermore, it was also reported that P3HT-*b*-PPerAcr with high molecular weights (*M*_n_ ~ 30,000) could form clear lamellar and cylindrical nanostructures, even in the melting state [37].

**Figure 5.**
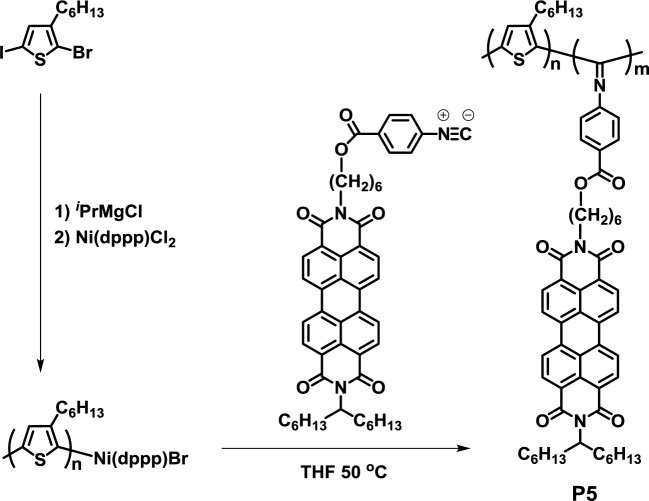
Structure of **P5**.

Recently, Bielawski *et al.* reported a useful one-pot synthesis of crystalline-crystalline D-A block copolymer, poly(3-hexylthiophene)-*b*-poly(perylene bisimide-functionalized-isocyanide) (**P5**), using a single nickel catalyst (Figure 5) [38]. The polymerization proceeded in a controlled fashion to give molecular weights that were proportional to the feed ratios of the monomers and catalyst. Furthermore, AFM observation exhibited that both the donor and acceptor components of the block copolymer self-stacked into lamellar nanostructures in the solid state.

**Figure 6.**
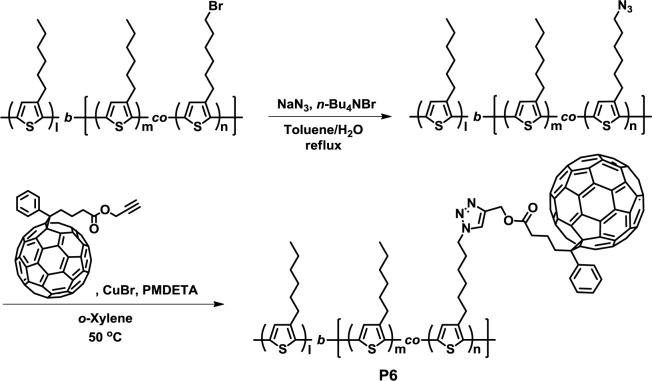
Synthesis of **P6**.

In 2012, Tajima *et al.* synthesized the regioregular polythiophene-based diblock copolymers with pendant fullerene units (**P6**) (Figure 6) [39]. Precursor diblock copolymers were synthesized via GRIM polymerization using 3-hexylthiophene and 3-(6-bromohexyl)thiophene, followed by the conversion of the bromide to the azide group. Then, the fullerene-attached polythiophene-based block copolymers (**P6**) were obtained via a click reaction between the precursor diblock copolymer and the alkyne-functionalized fullerene derivative. The ^1^H NMR analysis revealed the complete incorporation of fullerene derivatives. It was observed from the X-ray diffraction (XRD) results that the **P6** and P3HT:PCBM physical mixture exhibited similar diffraction patterns, suggesting that the lamellar nanostructure and π-π stacking crystalline structure of **P6** were similar to those of P3HT.

## 3. Block Copolymers Composed of Donor and Acceptor Main Chain Blocks

**Figure 7.**
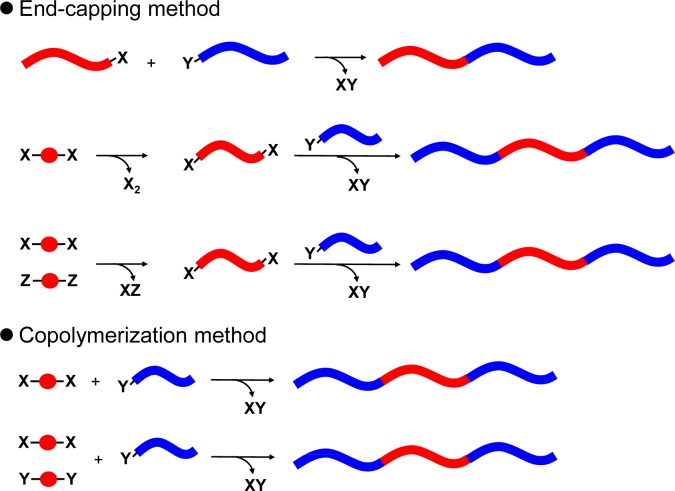
End-capping method and copolymerization method.

Vinyl or acrylate-based monomers usually applied for living chain-growth polymerization and various click chemistry, were utilized for the synthesis of D-A block copolymers with acceptor pedant units. However, most of these polymers have aliphatic segments in the polymer backbone, which can function as insulators in solar cells. Accordingly, a polymer architecture where donor and acceptor main chain blocks are directly connected without insulator units (fully conjugated D-A block copolymers) was considered to be ideal for solar cell applications. The method for synthesizing fully conjugated D-A block copolymer is classified in the two methods, which are the end-capping method and the copolymerization method (Figure 7).

### 3.1. End-Capping Method

In the end-capping method, fully conjugated D-A block copolymers are generally prepared by a two-step reaction. Donor and acceptor blocks with their respective end-functional groups are prepared in the first reaction, and the second reaction is carried out between donor and acceptor blocks to yield fully conjugated block copolymers.

**Figure 8.**
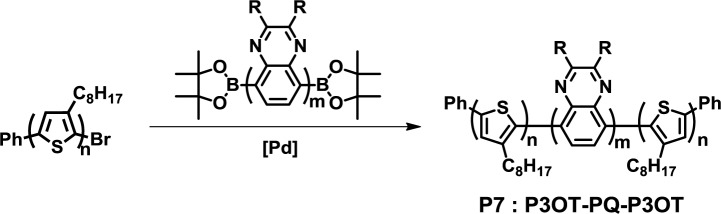
Synthesis of **P7**.

The synthesis of fully conjugated D-A-D block copolymers by Collard *et al.* is a typical example of the end-capping method (Figure 8) [40]. Poly(3-octylthiophene) with a monobromo-functional group and poly(quinoline) with diboronic ester functional groups were synthesized via GRIM polymerization and the Yamamoto coupling reaction, respectively. Then, a Suzuki coupling reaction between poly(3-octylthiophene) and poly(quinoxaline) gave poly(3-octylthiophene)-*block*-poly(quinoxaline)-*block*-poly(3-octylthiophene) (**P7**). The purification of **P7** was carried out with the Soxhlet extraction using methanol, acetone and hexane to remove the unreacted starting materials.

**Figure 9.**
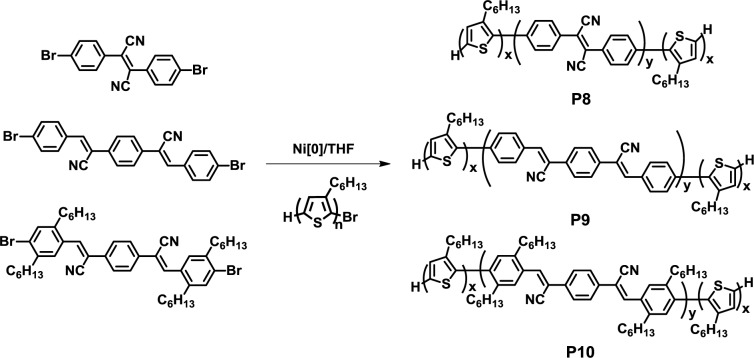
Synthesis of **P8**–**P10**.

In 2006, Scherf *et al.* reported a facile one-pot synthesis of fully conjugated D-A-D triblock copolymers composed of a poly(3-hexylthiophene) donor and cyano-substituted poly(phenylenevinylene) acceptor blocks (**P8**–**P10**) (Figure 9) [41]. Here, cyano-substituted dibromo monomers were polymerized for 8–14 h in the presence of nickel catalyst under the Yamamoto coupling reaction, and regioregular P3HT was then added as the end-capping agent to yield the desired triblock copolymers. AFM observation revealed the formation of nanosized spherical aggregates with a diameter of 60–90 nm, which were derived from the crystallization of poly(3-hexylthiophene) blocks, whereas the P3HT/acceptor homopolymer physical mixture exhibited irregular nanostructures with larger domains compared to the block copolymers. Considering the short exciton diffusion lengths of semiconducting materials, the observed nanomorphology behavior of the block copolymer can be used in solar cell applications.

**Figure 10.**
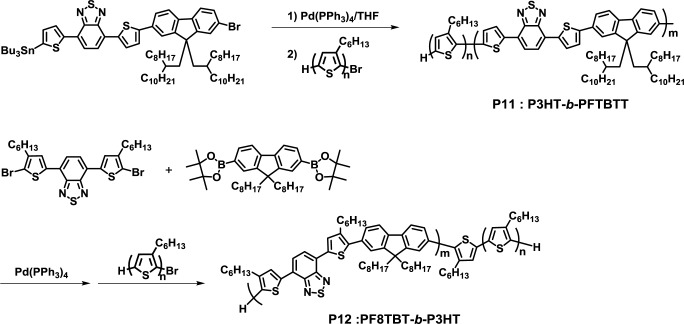
Synthesis of **P11** and **P12**.

Greenham and Scherf presented the synthesis of a fully conjugated D-A diblock copolymer (P3HT-*b*-PFTBTT, **P11**) by a one-step Stille coupling reaction, which was the self-condensation of PFTBTT monomer, followed by the addition of P3HT as the end-capping agents (Figure 10) [42]. Furthermore, **P11** exhibited clear lamellar nanomorphology when annealed at 220 °C for 2 h.

Sommer *et al.* also reported a one-pot synthesis of a fully conjugated D-A diblock copolymer (PF8TBT-*b*-P3HT, **P12**) based on the Suzuki coupling reaction [43]. Despite the fact that it is difficult to separate desired block copolymers and excess end-capping agents by the one-step method, the group successfully obtained pure **P12** by purification with preparative gel permeation chromatography (GPC). However, **P12** did not exhibit clear phase separation in the nano scale; owing to the presence of two side chains at the thiophene rings in PF8TBT, the solubility of P3HT in PF8TBT was enhanced. This idea can be used for designing fully conjugated D-A block copolymers that are capable of forming a well-defined phase separation.

### 3.2. Copolymerization Method with an End-Functional Polymer

In this method, regioregular P3HT synthesized by GRIM polymerization was commonly used as the end-functional polymer. The synthesis of fully conjugated D-A block copolymers was prepared in a one-step reaction by adding P3HT and monomers for the acceptor blocks at the same time.

**Figure 11.**
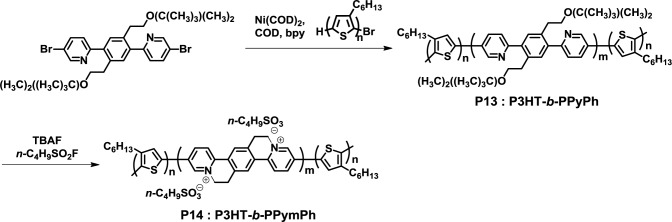
Synthesis of **P13** and **P14**.

In 2011, Swager *et al.* synthesized a fully conjugated D-A-D triblock copolymer (P3HT-*b*-PPyPh, **P13**) from P3HT and 6,6’-(2,5-bis(2-(tert-butyldimethylsilyloxy)ethyl)-1,4-phenylene)bis(3-bromopyridine) in the presence of nickel catalyst under Yamamoto coupling reaction conditions (Figure 11) [44]. The desired block copolymer with narrow polydispersity (1.44) was successfully obtained by this method; however, a small amount of byproduct (e.g., coupled-P3HT and PPyPh homopolymer) could not remove from the obtained products. Interestingly, **P13** was completely converted to P3HT-*b*-PPymPh (**P14)** via a cyclization process using tetra-*n*-butylammonium fluoride and nonafluorobutanesulfonyl fluoride as cyclization reagents. Furthermore, nanodomains of *ca.* 20 nm diameter were observed in **P14** using TEM.

**Figure 12.**
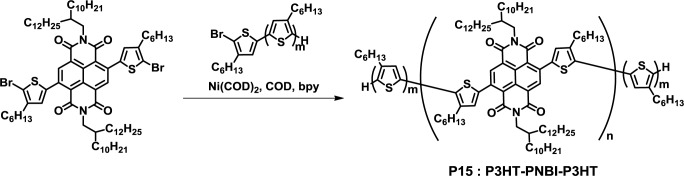
Synthesis of **P15**.

Nakabayashi *et al.* also demonstrated the synthesis of a conjugated D-A-D triblock copolymer with a naphthalene bisimide (NBI)-based acceptor block (**P15**) (Figure 12) [45]. The desired polymer with narrow polydispersity (1.28) was obtained in a high yield. Furthermore, **P15** exhibited a wide light absorption in the range of 400–900 nm by a thermal annealing treatment. The observed wide light absorption behavior can be an advantage for solar cell applications. Recently, the thin film morphology of a P3HT/**P15** blend film was studied using grazing-incidence wide-angle X-ray scattering (GIWAXS) analysis. The optimization of thin film fabrication conditions resulted in π-π stacking nanomorphology, where the P3HT homopolymer and P3HT blocks were arranged predominantly in the edge-on manner, and poly(naphthalene bisimide) (PNBI) blocks were arranged predominantly in the face-on orientation onto the substrate.

**Figure 13.**
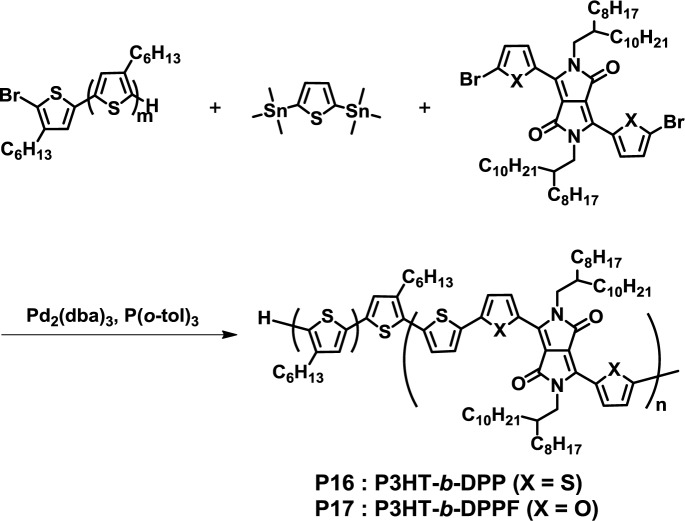
Synthesis of **P16** and **P17**.

In 2012, Hawker *et al.* reported the successful synthesis of fully conjugated D-A diblock copolymers composed of a P3HT donor and diketopyrrolopyrrole (DPP)-based acceptor blocks under Stille coupling reaction conditions (**P16**) (Figure 13) [46]. In this method, several block copolymers with the different D-A block compositions were obtained in quite high yields (>90%). AFM and GIWAXS analyses revealed that **P16** formed well-ordered nanostructures with a domain length of *ca.* 30 nm. The covalent bond between the donor and acceptor blocks resulted in clear microphase separation without any aggregation.

**Figure 14.**
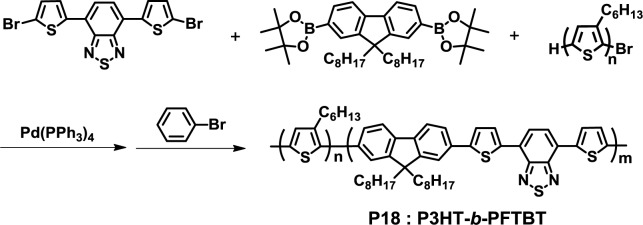
Synthesis of **P18**.

In 2013, Verduzco *et al.* synthesized a fully conjugated D-A diblock copolymer (P3HT-*b*-PFTBT, **P18**) by the Suzuki coupling reaction (Figure 14) [47]. Detailed XRD and GIWAXS analyses demonstrated that **P18** resulted in well-defined lamellar phase separation with edge-on orientation by optimal thermal annealing, which is ideal for an efficient photoelectric conversion process. The absence of two side chains at the thiophene rings in PFTBT might lead to the phase separation between two blocks, as mentioned previously by Sommer *et al.* [43].

**Figure 15.**
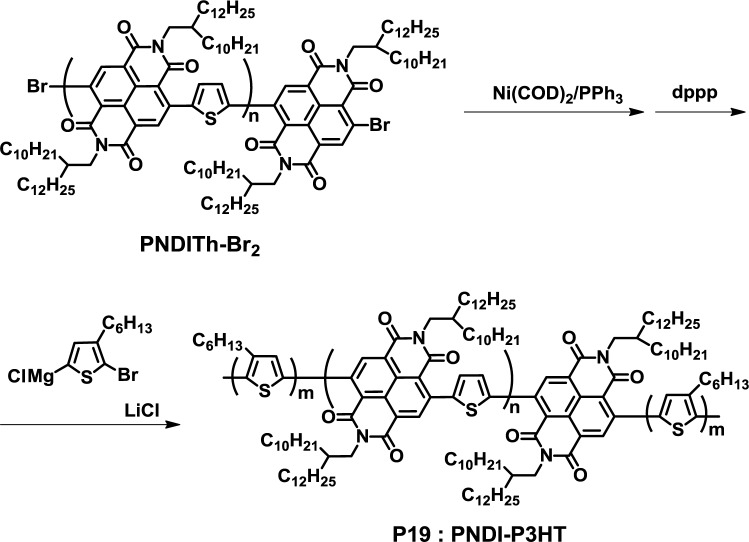
Synthesis of **P19**.

Recently, Higashihara *et al.* developed an interesting approach for the synthesis of fully conjugated D-A block copolymers (Figure 15) [48]. In this method, a bromo-terminated NBI-based polymer (PNDITh-Br_2_) was firstly synthesized by the Stille coupling reaction, which was then transferred to a macroinitiator with Ni(COD)_2_ and 1,3-bis(diphenylphosphino)propane (dppp). Using this macroinitiator, a thiophene monomer was polymerized by GRIM polymerization to yield fully-conjugated D-A-D triblock copolymers (PNDI-P3HT, **P19**). The obtained polymer exhibited a unimodal size-exclusion chromatography (SEC) curve and narrow polydispersity (1.15), indicating the transformation to the macroinitiator, followed by the GRIM polymerization of the thiophene monomer, which was successfully achieved.

As for the synthesis of fully conjugated D-A block copolymers, the removal of impurities (e.g., unreacted oligomers) from the desired block copolymers is quite difficult; thus, a small amount of impurities remains in block copolymers. Nevertheless, their unique characteristics, especially their self-assembly behaviors, are still attractive for solar cell applications.

## 4. All Polymer Solar Cells Using D-A Block Copolymers

One of challenging applications of D-A block copolymers is their use as fullerene alternative material in OPVs (Table 1). In 2012, Nakabayashi *et al.* reported all-polymer solar cells with P3HT/**P15** blend active layers [45]. In their report, the PCE was strongly dependent on the thermal annealing of active layers, as it contributed to the improvement of light absorption behavior of **P15** and morphological change in the blend active layers; a PCE of 1.28% was achieved by optimal thermal annealing, whereas a PCE of the as-spun blend active layer was 0.37%. Recently, detailed GIWAXS analysis revealed that the thermal-annealed P3HT/**P15** blend active layers induced the formation of more π-π stacked nanostructures compared to the as-spun blend active layer. Finally, the further optimization of device fabrication conditions resulted in the PCE of 1.60% in the P3HT/P**15** system [49].

Furthermore, several research groups reported single-component active-layer OPVs using D-A block copolymers. In 2007, Thelakkat *et al.* reported OPVs using D-A block copolymers, fabricated from **P1**–**P3** as a single-component active layer. PCEs of 0.052%–0.323% were achieved in those OPVs [33]. The morphology behavior was strongly dependent on the molecular weight of the block copolymer in their system, and the difficulty in forming a proper morphology resulted in the extremely poor PCEs.

OPVs fabricated from single-component active layers were also reported using **P6** and **P18**, respectively. Using optimized thermal annealing and film thickness conditions, an open-circuit voltage (*V*_oc_) of 0.48 V, a short-current (*J*_sc_) of 8.14 mA/cm^2^ and a fill factor (*FF*) of 0.63 were obtained, which resulted in a PCE of 2.46% in the **P6** system [39]. As mentioned earlier, a single **P6** layer could form nano phase separation similar to that of the P3HT/PCBM physical mixture, which resulted in relatively high PCEs. As for **P18**, a PCE of 2.70% was achieved with a *V*_oc_ of 1.14 V, a *J*_sc_ of 5.00 mA/cm^2^ and an *FF* of 0.45 using the single **P18** active layer annealed at 165 °C for 10 min [50]. It was expected that the lamellar phase separation with the edge-on orientation would lead to an efficient photoelectric conversion process to yield the excellent PCE. Surprisingly, the observed *V*_oc_ was considerably high, and the causes for such a high *V*_oc_ are still under investigation. Those results indicated that the performance of all-polymer solar cells was strongly dependent on the nanomorphology in active layers; a well-defined nanostructure, such as the π-π stacked nanostructure and the lamellar nanostructure with the edge-on orientation, is the key to accomplishing high PCEs in all-polymer solar cells.

## 5. Concluding Remarks

In the past decade, the synthetic methodology of D-A block copolymers has been widely investigated by effectively utilizing GRIM polymerization, Stille coupling polymerization, Suzuki coupling polymerization, Yamamoto coupling polymerization, and so on. The self-assembly behavior of D-A block copolymers is also an interesting topic; because in some cases, a clear nanomorphology was obtained even in fully conjugated D-A block copolymers by tuning the polymer structure and thin film fabrication conditions. The self-assembly behavior of donor-acceptor block copolymers has been utilized in solar cell applications. To date, all-polymer solar cells using D-A block copolymers achieved PCEs of *ca.* 3%, which are comparable to that of P3HT/PCBM-based OPVs; however, their PCEs are still lower than those of fullerene-based OPVs.

We believe that advancements can be made in D-A block copolymers, including the development of novel synthetic methodologies, polymer architectures and self-assembly studies. Previous studies have shown that the formation of a suitable nanomorphology for an efficient photoelectric conversion process was the key to realize high-performance all-polymer solar cells. Accordingly, research on the polymer architecture and self-assembly relationship can be a promising strategy for high-performance all-polymer solar cells.

Furthermore, poly(3-alkylthiophene) is commonly used as donor blocks in most cases, which can limit the diversity of the design of acceptor blocks. For example, donor blocks with lower HOMO levels can be useful for a single-component solar cell application, because this might lead to a larger *V*_oc_, resulting in the improvement of the PCE. Donor blocks composed of the ring-fused structure are also promising, considering the enhancement of the light absorption behaviors. Thus, if promising donor blocks instead of poly(3-alkylthiophene) are discovered, the tuning of the polymer properties (e.g., light absorption properties, electrochemical properties, solubility and crystallinity) would be easier, which would also be helpful in finding the ideal D-A block copolymers, leading to the realization of high-performance all-polymer solar cells.

## Figures and Tables

**Table 1.** All-polymer solar cells using donor-acceptor block copolymers.

Block copolymer	Donor	PCE (%)	*V*_oc_ (V)/*J*_sc_ (mA/cm^2^)/*FF*	Ref.
**P1** ^a^	–	0.065	0.67/0.23/0.32	[33]
**P2** (low *M*_n_) ^a^	–	0.323	0.69/1.14/0.32	[33]
**P2** (high *M*_n_) ^a^	–	0.052	0.53/0.24/0.32	[33]
**P3** ^a^	–	0.262	0.53/1.21/0.31	[33]
**P6** ^a^	–	2.46	0.48/8.14/0.63	[39]
**P15**	P3HT ^b^	1.28	0.56/4.57/0.50	[45]
**P15**	P3HT ^b^	1.60	0.59/4.43/0.61	[49]
**P18** ^a^	–	2.70	1.14/5.00/0.45	[50]

a All-polymer solar cells with single-component active layers.

b P3HT (*M*_w_ = 50,000–70,000, *rr* = 91%–94%) was purchased from Rieke Metals.
